# Depression as a Cardiovascular Risk Marker in Pregnancy: Hypertensive and Arrhythmic Maternal Outcomes in a Retrospective Matched Cohort

**DOI:** 10.3390/jcm15113995

**Published:** 2026-05-22

**Authors:** Nilima Rajpal Kundnani, Adelina Mogos, Laurențiu Augustus Barbu, Gabriel Florin Răzvan Mogoș, Victor Buciu, Alexandru Caraba, Claudia Borza, Emil Florin Hut

**Affiliations:** 1 Department VI-Cardiology, “Victor Babes” University of Medicine and Pharmacy, 300041 Timisoara, Romania; 2 Research Centre of Timisoara Institute of Cardiovascular Diseases, “Victor Babes” University of Medicine and Pharmacy, 300041 Timisoara, Romania; 3 Doctoral School, University of Medicine and Pharmacy of Craiova, 200349 Craiova, Romania; 4 Railway Clinical Hospital, 200349 Craiova, Romania; 5 Department of Surgery, University of Medicine and Pharmacy of Craiova, 200349 Craiova, Romania; 6 Doctoral School, “Victor Babes” University of Medicine and Pharmacy, 300041 Timisoara, Romania; 7 Department of Internal Medicine, Diabetes and Systemic Rheumatology, “Victor Babes” University of Medicine and Pharmacy, 300041 Timisoara, Romania; 8 Discipline of Pathophysiology, Department of Functional Science, “Victor Babes” University of Medicine and Pharmacy, 300041 Timisoara, Romania; 9 Center for Translational Research and Systems Medicine, “Victor Babes” University of Medicine and Pharmacy, 300041 Timisoara, Romania; 10 Centre of Cognitive Research in Pathological Neuro-Psychiatry NEUROPSY-COG, “Victor Babes” University of Medicine and Pharmacy, 300041 Timisoara, Romania; 11 Department of Surgical Semiology, “Victor Babes” University of Medicine and Pharmacy, 300041 Timisoara, Romania

**Keywords:** antenatal depression, pregnancy, gestational hypertension, preeclampsia, arrhythmia, Holter monitoring, cardiovascular risk marker

## Abstract

**Background**: Antenatal depression has been associated with systemic inflammation, autonomic imbalance, and vascular dysfunction, yet its relationship with clinically relevant cardiovascular complications during pregnancy remains insufficiently defined. Objective: To evaluate whether antenatal depression diagnosed before cardiologic assessment is associated with gestational hypertension, preeclampsia, and clinically significant Holter-confirmed arrhythmias in a tertiary-care population of pregnant women referred for cardiology assessment. **Methods**: We conducted a retrospective secondary matched cohort analysis nested within a prospectively approved doctoral research protocol (approval no. 76/02.10.2023; approved study interval: 2 October 2023–10 February 2025), including deliveries from October 2023 to February 2025. During this 16-month interval, 12,436 deliveries were recorded. The index point was the first cardiology specialist evaluation performed between 22 + 0 and 36 + 6 weeks’ gestation. Pregnancies with a depressive disorder diagnosed by structured psychiatric interview (SCID-5) before cardiology evaluation were classified as exposed. Depression severity was categorized as mild (*n* = 44), moderate (*n* = 62), or severe (*n* = 24), and psychotropic medication class at index was recorded. Each depressed case was matched 1:3 with non-depressed controls by gestational age at index, calendar year, maternal age, BMI category, smoking status, and parity; adjusted models included BMI and psychotropic medication class. **Results**: The final referral-enriched cohort included 130 depressed pregnancies and 390 matched controls (*n* = 520), all of whom underwent cardiology evaluation. Between 22 + 0 and 36 + 6 weeks’ gestation, gestational hypertension occurred in 18.5% vs. 10.0% (*p* = 0.010), preeclampsia in 8.5% vs. 4.9% (*p* = 0.12), and clinically significant Holter-confirmed arrhythmias in 15.4% vs. 6.9% (*p* = 0.003) in depressed versus control groups, respectively. After adjustment, depression remained independently associated with gestational hypertension (aOR 1.85, 95% CI 1.12–3.05; *p* = 0.016) and arrhythmia (aOR 2.05, 95% CI 1.18–3.57; *p* = 0.011). A numerical, exploratory severity-response gradient was observed across mild, moderate, and severe depression strata, most clearly for Holter-confirmed arrhythmias; however, the severe-depression stratum was small (*n* = 24). **Conclusions**: Antenatal depression was associated with a modest but significant increase in gestational hypertension and clinically significant Holter-confirmed arrhythmias during late pregnancy among women referred for cardiology assessment. The higher preeclampsia rate in depressed pregnancies was not statistically significant. These findings support antenatal depression as a cardiovascular risk marker in gestation rather than proof of causality.

## 1. Introduction

Depressive disorders during pregnancy are common and clinically heterogeneous, spanning mild depressive episodes to severe or recurrent forms that require specialist care. Dadi et al. estimated a high global burden of antenatal depression, with prevalence varying substantially by setting, diagnostic method, and socioeconomic context [1]. Other systematic reviews have reported comparable prevalence ranges, supporting antenatal depression as a frequent clinical problem rather than a rare psychiatric comorbidity [2,3]. Because antenatal depression is often accompanied by measurable biological changes—including inflammatory activation with elevations in interleukin-6 (IL-6), C-reactive protein (CRP), and tumor necrosis factor-alpha (TNF-alpha), as well as autonomic dysregulation—it has increasingly been discussed as a condition with potential cardiovascular implications beyond its psychiatric burden [4,5,6]. Complementary evidence from drug-naive major-depression cohorts has shown that higher C-reactive protein can coexist with altered serum lipid profiles, reinforcing an inflammatory-metabolic interface through which depressive illness may intersect with cardiovascular risk [7].

Hypertensive disorders of pregnancy remain a leading cause of maternal and perinatal morbidity, with preeclampsia representing a core phenotype of pregnancy-related systemic vascular dysfunction. Recent global estimates based on systematic evidence synthesis suggest that preeclampsia affects roughly 4–5% of pregnancies worldwide, although rates vary by region and diagnostic criteria [8,9]. Contemporary mechanistic models describe preeclampsia as a placenta-driven disorder characterized by maternal endothelial injury and systemic vascular dysfunction, in part mediated by antiangiogenic factors and oxidative-inflammatory pathways [10,11,12]. In parallel, epidemiologic evidence has linked depressive symptoms measured before the onset of hypertension to a higher risk of hypertensive disorders of pregnancy, supporting temporality consistent with depression as a risk marker rather than solely a consequence of obstetric complications [13,14].

Arrhythmias are encountered with increased frequency during pregnancy because gestation combines hormonal effects on cardiac electrophysiology, sympathetic activation, heart-rate acceleration, and major hemodynamic loading [15,16,17]. Plasma volume expands by approximately 40–50%, increasing atrial and ventricular wall stress; this mechanical stretch may modulate stretch-activated ion channels and shorten the threshold for ectopy or re-entry in susceptible patients [15,17]. At a pathophysiological level, depressive disorders have been consistently associated with altered cardiac autonomic control, commonly measured by reduced high-frequency (HF) power, reduced global heart-rate variability, or an elevated low-frequency/high-frequency (LF/HF) ratio on HRV analysis [4,6,18]. In addition, depression has been linked with inflammation-associated endothelial dysfunction and cardiovascular disease in non-pregnant populations, and severity-dependent cardiovascular risk has been described outside pregnancy, offering a biologically coherent bridge between depressive illness, vascular reactivity, and arrhythmogenic vulnerability [5,19,20].

Ackerman-Banks et al. examined 119,422 pregnancies and reported increased risks of new cardiovascular conditions, including arrhythmia/cardiac arrest and new hypertension, among pregnancies complicated by prenatal depression [21]. However, population-level diagnostic codes cannot fully distinguish benign ectopy from clinically significant rhythm disturbances, and they do not provide the same temporal anchoring as a standardized cardiology assessment [22]. We therefore selected the 22 + 0 to 36 + 6 weeks’ gestational window because it begins after the diagnostic threshold for gestational hypertension and preeclampsia, overlaps with the period of maximal maternal volume expansion and autonomic adaptation, and ends before term/peripartum management introduces additional confounding. We hypothesized that antenatal depression documented before cardiology evaluation would be associated with higher risks of gestational hypertension and clinically significant Holter-confirmed arrhythmias, with a stronger association expected for arrhythmias than for preeclampsia because arrhythmogenesis is more directly linked to autonomic and electrophysiological vulnerability whereas preeclampsia requires a placenta-driven antiangiogenic pathway.

## 2. Methods

### 2.1. Study Design and Setting

We performed a retrospective secondary matched cohort analysis within a broader doctoral research project prospectively approved by the institutional Ethics Committee before initiation of the approved study interval. After completion of the approved study interval and database lock, maternal, psychiatric, cardiologic, and obstetric variables were extracted from standardized digital registries and analyzed retrospectively. During the 16-month study interval, 12,436 deliveries were recorded in the institution; this denominator refers to all deliveries during the approved interval, not to annual birth volume. The analytic cohort should therefore be interpreted as referral-enriched, because inclusion required cardiology evaluation rather than unselected obstetric follow-up.

The study was designed and reported in accordance with the Strengthening the Reporting of Observational Studies in Epidemiology (STROBE) statement for observational studies [23]. The index time point was defined as the first cardiology specialist evaluation performed between 22 + 0 and 36 + 6 weeks’ gestation. Exposure status and baseline characteristics were determined prior to or at the index visit to preserve temporal coherence between psychiatric diagnosis and cardiovascular evaluation.

The present cardiovascular analysis represents one component of a broader approved doctoral research project. The present manuscript is restricted to maternal hypertensive and clinically significant Holter-confirmed arrhythmic endpoints anchored to the late-pregnancy cardiology evaluation; neonatal and neurodevelopmental analyses will be reported separately and are not used as endpoints in this paper.

### 2.2. Exposure Definition

Exposure was defined as a documented psychiatric specialist diagnosis of depressive disorder recorded in the institutional electronic system prior to the index cardiology evaluation. Diagnoses were established by board-certified psychiatrists using the Structured Clinical Interview for DSM-5 Disorders (SCID-5), with diagnostic coding subsequently entered according to ICD-10 categories for depressive episodes and recurrent depressive disorder. Only diagnoses entered following formal specialist consultation were accepted as valid exposure.

Depression severity was extracted from psychiatric records and categorized as mild, moderate, or severe according to the SCID-5-based specialist assessment and the corresponding ICD-10 severity category entered in routine clinical documentation. These categories were treated as routine-practice clinical severity strata rather than psychometric score bands. For each exposed patient, we recorded depression subtype, severity stage, date of diagnosis, and psychotropic medication class at the time of cardiologic evaluation, including selective serotonin reuptake inhibitors (SSRIs), serotonin-norepinephrine reuptake inhibitors (SNRIs), antipsychotic augmentation, benzodiazepines, or absence of pharmacologic treatment. Medication dose and duration of use were not consistently available in the retrospective dataset and were therefore not analyzed.

The control group consisted of pregnant women with cardiology evaluation in the same gestational window and no documented psychiatric diagnosis of depressive disorder prior to the index visit. Thus, controls were not healthy pregnant women from the general obstetric population, but women who entered the same tertiary cardiology pathway without a documented depressive disorder. This design improves comparability of cardiovascular ascertainment but may introduce selection bias related to the reason for cardiology referral.

### 2.3. Inclusion and Exclusion Criteria

Eligible participants were women with singleton pregnancies who underwent cardiology specialist evaluation between 22 + 0 and 36 + 6 weeks’ gestation and had complete psychiatric and obstetric documentation available.

To isolate cardiovascular outcomes attributable to gestational physiology and depression status, we excluded women with chronic hypertension diagnosed prior to pregnancy or before 20 weeks’ gestation, pregestational diabetes mellitus, chronic kidney disease stage 3 or higher as defined by KDIGO criteria [24], known structural heart disease, previously documented arrhythmia prior to pregnancy, systemic autoimmune vascular disease, uncontrolled thyroid disease, or multiple gestation. Active smokers and women with caffeine intake were not excluded; smoking status was included in the matching procedure, whereas extreme caffeine intake and undocumented illicit substance use could not be reliably quantified from the retrospective records. These issues were retained as potential residual arrhythmogenic confounders.

### 2.4. Matching Procedure

Each depressed pregnancy was matched to three non-depressed controls using nearest-neighbor propensity score matching without replacement. Exact matching was performed on gestational age at the index cardiology visit within one week and calendar year of delivery. Propensity score estimation incorporated maternal age, body mass index category, smoking status, and parity. Socioeconomic status was not included because the hospital catchment and the analyzed cohort were socioeconomically homogeneous, with insufficient discriminatory variation for stable matching. Gravidity was also not included to avoid overfitting in a relatively small, exposed cohort and because parity was the more consistently encoded obstetric-history variable. A caliper width of 0.2 standard deviations of the logit of the propensity score was applied to ensure adequate covariate balance. Matching quality was assessed using standardized mean differences, with values below 0.1 considered indicative of acceptable balance.

### 2.5. Cardiology Assessment

All included patients underwent cardiology specialist consultation and standard 12-lead electrocardiography at the index visit. When ECG findings or symptoms suggested rhythm disturbance, 24 h Holter monitoring was performed; Holter assessment was therefore clinically triggered rather than universal. Cardiologists interpreting ECGs and Holter recordings were not blinded to psychiatric status because the analysis was retrospective and based on routine clinical documentation; this was considered a potential diagnostic-review bias. Blood pressure was measured according to routine institutional practice after seated rest, but the retrospective dataset did not uniformly identify whether each measurement was obtained using an automated oscillometric device or manual sphygmomanometer. This pathway may miss intermittent rhythm disturbances in patients with normal baseline ECGs and should be considered a potential detection bias.

### 2.6. Outcomes

The primary outcomes were gestational hypertension, preeclampsia, and Holter-confirmed arrhythmias. Gestational hypertension was defined as systolic blood pressure ≥ 140 mmHg and/or diastolic blood pressure ≥ 90 mmHg after 20 weeks’ gestation on at least two measurements obtained at least four hours apart, in accordance with the American College of Obstetricians and Gynecologists criteria [25,26]. Preeclampsia was defined as new-onset hypertension after 20 weeks’ gestation accompanied by proteinuria or evidence of end-organ dysfunction, also according to established obstetric guidelines [27,28]. Hypertensive outcomes were assessed from the index cardiology visit until delivery.

Arrhythmias were defined as supraventricular tachycardia, atrial fibrillation or flutter, non-sustained ventricular tachycardia, high-burden ectopy, multifocal premature ventricular complexes, or clinically relevant bradyarrhythmia documented during 24 h Holter monitoring performed in the index evaluation period. High-burden ectopy was defined as >500 premature ventricular complexes per 24 h, >1% of total beats, or multifocal premature ventricular complexes. Isolated low-burden supraventricular or ventricular ectopy was not counted as an arrhythmia endpoint.

Because individual rhythm subtypes were expected to be sparse, the primary arrhythmia endpoint was prespecified as a composite of clinically significant Holter-confirmed rhythm abnormalities. This approach increased endpoint stability but means that the adjusted odds ratio should be interpreted as the risk of any clinically significant arrhythmia, not as a subtype-specific estimate.

### 2.7. Statistical Analysis

Data entry and cleaning were performed using Microsoft Excel 2016 (Microsoft Corp., Redmond, WA, USA) [29]. Statistical analyses were conducted using IBM SPSS Statistics version 26 (IBM Corp., Armonk, NY, USA) [30] and R version 4.3.3 (R Foundation for Statistical Computing, Vienna, Austria) [31]. Continuous variables were assessed for normality using the Shapiro–Wilk test and are presented as mean +/− standard deviation or median with interquartile range as appropriate. Categorical variables are presented as counts and percentages.

Between-group comparisons were performed using Student’s *t*-test or Mann–Whitney U test for continuous variables and chi-square or Fisher’s exact test for categorical variables. Conditional logistic regression models were constructed to estimate crude and adjusted odds ratios with 95% confidence intervals for each outcome, accounting for the matched design. Adjustment variables included body mass index and psychotropic medication class at the time of cardiologic evaluation. Severity-response patterns across mild, moderate, and severe depression were evaluated using the Cochran-Armitage test for trend. Two-sided *p*-values below 0.05 were considered statistically significant; a one-sided directional trend *p*-value was additionally reported for the arrhythmia severity analysis because an increasing severity gradient was specified a priori. Comorbid anxiety disorders and sleep disturbance were not available as consistently structured variables in the retrospective database and therefore could not be included in the propensity score or conditional logistic regression models.

### 2.8. Ethical Considerations

The study was conducted in accordance with the Declaration of Helsinki [32]. The present manuscript is a retrospective secondary analysis of the cardiovascular component of that approved protocol, performed after completion of the approved data-collection interval. Data handling complied with the General Data Protection Regulation (EU 2016/679) [33].

## 3. Results

### 3.1. Cohort Derivation

During the approved study interval, from October 2023 to February 2025, a total of 12,436 deliveries were recorded in our institution. During this period, 348 pregnant women had a psychiatric specialist diagnosis of depressive disorder documented in the electronic system prior to 37 weeks’ gestation. Of these, 156 underwent cardiology evaluation between 22 + 0 and 36 + 6 weeks. After exclusions, 130 depressed pregnancies met full eligibility criteria. Exact exclusion reasons should be reported from the source database as follows before resubmission: chronic hypertension (*n* = 8), structural heart disease (*n* = 3), prior arrhythmia (*n* = 8), chronic kidney disease stage 3 or higher (*n* = 1), pregestational diabetes (*n* = 2), autoimmune vascular disease (*n* = 2), uncontrolled thyroid disease (*n* = 2).

Each exposed case was matched 1:3 with non-depressed controls who underwent cardiology evaluation in the same gestational window, resulting in a final analytic cohort of 520 pregnancies (130 depressed and 390 controls).

### 3.2. Baseline Characteristics

Baseline maternal characteristics after matching are presented in Table 1. Groups were well balanced with standardized mean differences below 0.1 for all matching variables.

Continuous variables are presented as mean +/− standard deviation. Categorical variables are presented as number (percentage). Matching was performed using nearest-neighbor propensity score matching with exact matching on gestational age at index (+/−1 week) and calendar year. Depression-severity and psychotropic-treatment rows are descriptive variables for the exposed group only and were added to improve transparency regarding psychiatric phenotype and treatment at the index evaluation.

Among depressed patients, 44 (33.8%) were classified as mild, 62 (47.7%) as moderate, and 24 (18.5%) as severe depression. SSRI therapy at index was documented in 84 patients (64.6%). Complete class-level medication counts should be verified in the source database before resubmission because dose and duration were not consistently available.

## 4. Referral Pathway and Holter Monitoring Use

Because both exposed and control patients underwent cardiology evaluation, the cohort represents a tertiary referral-enriched population rather than an unselected obstetric population. The main indications for cardiology assessment should be reported descriptively to clarify whether depressed and non-depressed patients entered the cardiology pathway for broadly similar reasons.

Referral indications should be extracted descriptively from the source database before final resubmission. Recommended categories are palpitations or perceived rhythm disturbance, persistent sinus tachycardia, abnormal baseline 12-lead ECG, elevated blood pressure or suspected hypertensive disorder, dyspnea or chest discomfort, syncope or presyncope, and other or combined indications. 

Because these categories may overlap, they should be reported as non-mutually exclusive unless the database contains a single primary referral indication for each patient.

Holter monitoring was not performed universally but was triggered by symptoms or baseline ECG findings. This selective approach increased clinical specificity but may have missed intermittent arrhythmias in patients with normal baseline ECGs.

### 4.1. Primary Outcomes

Cardiovascular outcomes are summarized in Table 2.

Arrythmia subtipes diagnosed trough Holter-monitoring is better visualized in Supplementary Table S1. 

Gestational hypertension and preeclampsia were assessed from index cardiology evaluation until delivery. Arrhythmias were defined as clinically significant rhythm disturbances confirmed by 24 h Holter monitoring following abnormal ECG.

Gestational hypertension occurred significantly more frequently in depressed pregnancies compared with controls. Clinically significant Holter-confirmed arrhythmias were also significantly more common in the depressed group. Although preeclampsia occurred more frequently among depressed patients, the difference was not statistically significant and is not interpreted as a trend.

**Figure 1:**
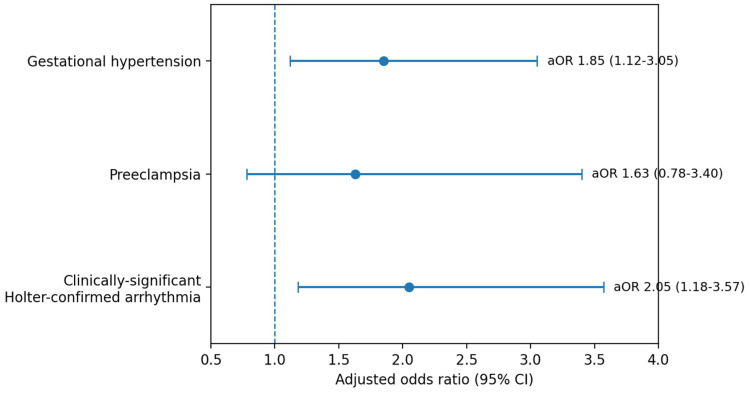
Forest plot of adjusted odds ratios for cardiovascular outcomes associated with antenatal depression, with exact point estimates and 95% confidence intervals.

The distribution of cardiovascular outcomes between exposed and control groups is illustrated in Figure 1.

Adjusted odds ratios were estimated using conditional logistic regression accounting for the matched design and adjusted for body mass index and psychotropic medication class at index evaluation. Error bars show 95% confidence intervals, and numerical labels provide the exact adjusted odds ratio and confidence interval for each endpoint. Clinically significant Holter-confirmed arrhythmias excluded isolated low-burden ectopy.

### 4.2. Multivariable Analysis

Conditional logistic regression results are presented in Table 3.

Conditional logistic regression accounting for the matched design. Adjusted models included body mass index and psychotropic medication class at index evaluation. Crude odds ratios are shown to demonstrate the degree of attenuation after adjustment.

Antenatal depression remained independently associated with gestational hypertension and clinically significant Holter-confirmed arrhythmias after adjustment. Adjustment for BMI and psychotropic medication class modestly attenuated the crude estimates but did not abolish the associations for gestational hypertension or arrhythmia. The confidence interval for arrhythmia remained relatively wide (1.18–3.57), indicating limited endpoint precision and the need for larger cohorts to refine this estimate. The association with preeclampsia remained non-significant.

### 4.3. Outcomes by Depression Severity

A graded increase in cardiovascular events was observed across depression severity strata. These data are summarized in Table 4.

Severity defined according to SCID-5-based psychiatric specialist documentation prior to index cardiology evaluation. *p* for trend was calculated using the Cochran-Armitage test across mild, moderate, and severe categories. For arrhythmia, the prespecified one-sided directional *p* for trend was 0.041; the two-sided *p*-value is shown in the table.

The proportion of events increased numerically from mild to severe depression. Formal two-sided trend testing did not reach statistical significance for gestational hypertension or preeclampsia and was borderline for clinically significant Holter-confirmed arrhythmia; therefore, the severity analysis should be interpreted as exploratory. The severe-depression subgroup contained only 24 patients, making the apparent stepwise pattern unstable and unsuitable as definitive dose–response evidence.

## 5. Discussion

### 5.1. Antenatal Depression and Hypertensive Disorders of Pregnancy: Pathophysiological Links

Antenatal depression is increasingly conceptualized as a systemic condition characterized by neuroendocrine and autonomic dysregulation, rather than a purely psychological diagnosis. Dysregulation of the hypothalamic–pituitary–adrenal (HPA) axis and altered autonomic balance, particularly reduced parasympathetic tone with relative sympathetic predominance, have been consistently described in perinatal depression [34]. In pregnant populations, depressive symptoms have been associated with measurable alterations in heart rate variability, reflecting impaired vagal modulation and heightened sympathetic drive [35,36]. Because pregnancy itself induces substantial hemodynamic adaptation, sustained sympathetic activation may amplify vascular tone and blood pressure reactivity, increasing vulnerability to gestational hypertension [35].

Depression has also been linked to inflammatory activation and endothelial dysfunction in cardiovascular research, providing a second mechanistic axis that converges with hypertensive disease pathways [5]. Evidence in pregnant cohorts suggests that prenatal depression influences maternal physiological adaptation beyond subjective symptoms, supporting a systemic biological impact [37].

In contrast, preeclampsia is primarily regarded as a placenta-driven disorder characterized by anti-angiogenic imbalance and maternal endothelial injury. Increased circulating soluble fms-like tyrosine kinase-1 (sFlt-1) and soluble endoglin (sEng), together with reduced placental growth factor (PlGF), play a central role in generating the systemic endothelial dysfunction that defines the syndrome [38]. Contemporary syntheses further emphasize placental hypoxia–ischemia and oxidative stress as upstream triggers of this anti-angiogenic cascade [11,39].

This distinction may explain why, in our cohort, depression showed a clearer association with gestational hypertension than with overt preeclampsia. Depression-related autonomic and inflammatory activation may increase vascular reactivity and blood pressure instability without necessarily initiating the specific placental anti-angiogenic pathway required for the full preeclampsia phenotype [38,40,41,42]. However, the statistical interpretation is equally important: with only 130 depressed pregnancies and 30 total preeclampsia events, the analysis was likely underpowered to exclude a clinically meaningful difference, and a type II error remains possible.

Large-scale epidemiologic data support this framework. A nationwide cohort analysis demonstrated increased risks of newly diagnosed hypertension and arrhythmia among pregnancies complicated by prenatal depression, reinforcing the concept of depression as a cardiovascular vulnerability state during gestation [21].

### 5.2. Depression and Arrhythmogenic Vulnerability in Pregnancy

Pregnancy induces marked electrophysiological and hemodynamic adaptations, including increased heart rate, plasma volume expansion, and hormonal modulation of cardiac ion channels, all of which may lower arrhythmogenic thresholds [15,17,43,44]. While many pregnancy-associated arrhythmias are benign, clinically relevant rhythm disturbances are well documented, particularly in women referred for cardiology assessment [45,46,47].

Emotional and psychiatric disturbances during pregnancy may also intersect with rarer acute cardiovascular entities. Pregnancy-associated Takotsubo syndrome is uncommon, but recent narrative evidence indicates that it may occur during pregnancy or the immediate postpartum period and may carry maternal and fetal risk, especially when triggered by intense emotional, obstetric, or physiological stress [48,49]. Although Takotsubo syndrome was not an endpoint in the present cohort, it reinforces the broader principle that depressive and stress-related states can overlap with pregnancy-specific cardiovascular vulnerability [50].

Depressive disorders are consistently associated with autonomic imbalance, characterized by reduced heart rate variability and diminished vagal tone, reflecting relative sympathetic predominance [6,51]. Autonomic dysregulation influences myocardial repolarization and electrical stability, creating a substrate for supraventricular and ventricular arrhythmias. In parallel, depression has been linked to inflammatory activation, which can further affect ion channel function and myocardial conduction [5,52,53].

The overlap between pregnancy-related sympathetic activation and depression-associated autonomic imbalance offers a biologically coherent explanation for the increased risk of clinically significant Holter-confirmed arrhythmias observed in our cohort. Nevertheless, reverse causation cannot be excluded: palpitations or rhythm symptoms may have intensified anxiety and depressive symptoms and increased the likelihood of psychiatric referral or specialist diagnosis before cardiology evaluation. The retrospective design cannot fully separate depression as a pre-existing biological vulnerability from depression documented during a symptomatic cardiovascular trajectory.

Population-level evidence supports this relationship. A large nationwide cohort study demonstrated that prenatal depression was associated with increased risk of arrhythmia and other cardiovascular conditions during pregnancy [21]. Our findings reinforce this signal using objectively confirmed arrhythmia endpoints within a standardized cardiology framework. Because the Holter endpoint pooled heterogeneous rhythm disorders, the association should be interpreted as a signal for increased overall arrhythmia vulnerability rather than evidence that depression preferentially increases one specific arrhythmia subtype.

### 5.3. Medication Effects and the Depression–Cardiovascular Interface

Interpretation of the observed associations must consider psychotropic exposure, particularly selective serotonin reuptake inhibitors (SSRIs), which are frequently prescribed in moderate-severe antenatal depression. A comprehensive meta-review synthesizing data from PubMed and Web of Science highlighted that many reported risks attributed to antidepressants during pregnancy are strongly influenced by confounding by indication and illness severity, underscoring the difficulty of separating the biological effect of severe depression from the potential electrophysiological or vascular effects of higher-dose or longer-duration treatment [54]. In the present dataset, medication class was available, but exact dose and duration were not consistently captured.

With respect to hypertensive disorders, available evidence remains heterogeneous. A recent meta-analysis evaluating SSRI exposure and risk of preeclampsia or gestational hypertension concluded that associations are inconsistent and likely influenced by underlying psychiatric severity rather than medication alone [55,56,57,58]. Given that depressive severity correlates with greater autonomic and inflammatory activation [5], disentangling pharmacologic from disease-driven vascular effects is challenging.

Arrhythmic risk is biologically plausible because certain SSRIs, particularly citalopram, are associated with modest QTc prolongation, although clinically significant malignant arrhythmias remain rare in structurally normal hearts [59,60]. Case-based analyses confirm that torsades de pointes is uncommon and usually occurs in the presence of additional risk factors [61,62]. In pregnancy, where sympathetic tone is already elevated, small medication-related electrophysiological shifts could theoretically interact with gestational autonomic changes [17]. Because baseline 12-lead ECGs and SSRI exposure data were available, future analyses should specifically evaluate QTc interval, SSRI subtype, dose, and treatment duration in relation to Holter-confirmed rhythm outcomes.

Neonatal effects are more consistently documented. Late-pregnancy SSRI exposure has been associated with delayed neonatal adaptation and transient autonomic and respiratory symptoms, often dose-dependent [63,64]. Persistent pulmonary hypertension of the newborn (PPHN) has also been linked to SSRI exposure after mid-pregnancy in large population-based studies and meta-analyses, although absolute risk remains low, approximately 2–3 per 1000 exposed births [65,66,67]. Experimental and translational work confirms placental transfer of antidepressants and potential modulation of fetoplacental serotonergic signaling, while emphasizing generally small absolute teratogenic risks [68,69].

In our cohort, adjustment for medication class did not abolish the association between depression and cardiovascular outcomes, supporting the interpretation that the underlying depressive disorder—rather than pharmacotherapy alone—likely represents the dominant cardiovascular vulnerability mechanism, with medication acting as a potential modifier rather than a primary driver of risk.

## 6. Limitations

This study has several limitations. The retrospective design precludes causal inference, and residual confounding remains a central limitation despite matching and adjustment. Psychosocial factors such as socioeconomic status and chronic stress, which are closely linked to perinatal depression, could not be fully quantified; however, socioeconomic status was not included in the propensity score because the analyzed tertiary-care cohort was relatively homogeneous. Smoking was matched, but active smokers were not excluded, and extreme caffeine intake or undocumented illicit substance use could not be reliably quantified. Psychotropic medication dose and duration were not consistently captured, limiting separation of illness severity from treatment effects. In addition, comorbid anxiety disorders and sleep disturbance were not captured as consistently structured variables and could not be included in the adjusted models, although both may influence depressive status, cardiovascular symptoms, and referral for specialist assessment. The adjusted models were intentionally parsimonious, but they could not account for several clinically relevant factors, including prior anxiety disorders, sleep disturbance, chronic stress, detailed obstetric history beyond parity, and antidepressant dose or duration.

Restriction to women undergoing cardiology evaluation between 22 + 0 and 36 + 6 weeks strengthened outcome ascertainment—particularly for clinically significant Holter-confirmed arrhythmias—but may limit generalizability and introduce substantial referral bias. This cohort represents women referred for cardiology assessment in a tertiary setting; therefore, the absolute arrhythmia rate of 15.4% in depressed pregnancies should not be interpreted as a population prevalence and is likely inflated compared with an unselected obstetric population. ECG and Holter interpreters were not blinded to psychiatric status, which may have introduced review or ascertainment bias. In addition, because Holter monitoring was not applied to every participant, arrhythmia ascertainment depended on clinical suspicion, symptoms, or baseline ECG findings. This may have underestimated intermittent rhythm disorders in both groups and may also have amplified differences if depressed patients were more likely to report palpitations or anxiety-related cardiovascular symptoms.

Depression severity was based on structured SCID-5 psychiatric assessment documented in specialist records, but standardized longitudinal depression scores were not uniformly available. Mechanistic biomarkers central to hypertensive disorders—such as sFlt-1, PlGF, soluble endoglin, and inflammatory markers such as CRP, IL-6, and TNF-alpha—were not available, limiting causal physiological mapping between depressive illness, inflammation, angiogenic imbalance, and cardiovascular endpoints [38]. Blood-pressure device type was not uniformly encoded in the retrospective dataset, adding another measurement-standardization limitation. Although SCID-5-based clinical documentation provides stronger diagnostic validity than symptom screening alone, ICD-10 mild, moderate, and severe categories recorded in routine practice may be less homogeneous than EPDS or HAM-D thresholds; therefore, the severity-response analysis should be interpreted as exploratory and hypothesis-generating rather than definitive dose–response proof.

Moreover, neonatal outcomes and infant neurodevelopmental measures were not analyzed as endpoints within the present cardiovascular dataset, precluding any direct inference regarding downstream child development in this report.

### Future Perspectives

Future studies should integrate psychiatric phenotyping with cardiovascular biomarkers. Prospective designs incorporating standardized depression scales, heart-rate variability metrics, QTc analysis, angiogenic markers (sFlt-1/PlGF), and inflammatory panels (CRP, IL-6, TNF-alpha) could clarify whether depression primarily amplifies autonomic reactivity, inflammatory signaling, medication-related electrophysiological effects, or placental dysfunction. Given the central role of angiogenic imbalance in preeclampsia [38,42], examining whether depressive severity correlates with angiogenic profiles may explain differential associations with gestational hypertension versus preeclampsia.

Advanced electrophysiological assessment may further define arrhythmogenic vulnerability, as autonomic dysfunction is a consistent biological correlate of depressive disorders [6]. Large-scale cohort data already suggest that prenatal depression identifies women at increased cardiovascular risk during pregnancy [21]. Prospective interventional research evaluating whether optimized psychiatric management, QTc surveillance in selected SSRI-exposed patients, or targeted cardiovascular surveillance mitigates hypertensive or arrhythmic events would provide clinically meaningful next steps. As this manuscript forms part of a broader doctoral framework, future thesis components may examine neonatal and neurodevelopmental trajectories; however, such analyses should remain clearly separated from the maternal cardiovascular endpoints reported here and should be interpreted only when direct child-outcome data are available.

## 7. Conclusions

In this retrospective secondary matched cohort analysis nested within a prospectively approved doctoral protocol, antenatal depression diagnosed by structured psychiatric interview before late-pregnancy cardiologic evaluation in a referral-enriched tertiary cardiology cohort was associated with a modest but significant increase in gestational hypertension and clinically significant Holter-confirmed arrhythmias. The association with preeclampsia was not statistically significant, and the study was likely underpowered for this endpoint. These findings support antenatal depression as a cardiovascular risk marker during pregnancy among women referred for cardiology assessment, particularly for hypertensive surveillance and rhythm evaluation in symptomatic or severe cases. Based on the present data, routine Holter monitoring is not justified for all pregnant women with depression; however, obstetricians should consider at least a baseline 12-lead ECG and cardiology referral for women with severe depression, SSRI exposure with QTc concern, palpitations, syncope, persistent tachycardia, hypertension, or other cardiovascular symptoms.

## Figures and Tables

**Table 1:** Baseline characteristics of the matched cohort.

Variable	Depressed (*n* = 130)	Controls (*n* = 390)	*p*-Value
Maternal age (years)	31.2 ± 4.8	30.9 ± 4.6	0.58
BMI (kg/m^2^)	26.8 ± 4.3	26.4 ± 4.1	0.41
Nulliparity	52 (40.0%)	154 (39.5%)	0.93
Smoking during pregnancy	24 (18.5%)	66 (16.9%)	0.67
Gestational age at index (weeks)	29.4 ± 3.8	29.3 ± 3.6	0.84
Depression severity: mild	44 (33.8%)	Not applicable	Not applicable
Depression severity: moderate	62 (47.7%)	Not applicable	Not applicable
Depression severity: severe	24 (18.5%)	Not applicable	Not applicable
SSRI therapy at index	84 (64.6%)	Not applicable	Not applicable
SNRI therapy at index	18 (13.8%)	Not applicable	Not applicable
Benzodiazepine use at index	16 (12.3%)	Not applicable	Not applicable
Antipsychotic augmentation at index	8 (6.2%)	Not applicable	Not applicable
No psychotropic medication at index	22 (16.9%)	Not applicable	Not applicable

**Table 2:** Cardiovascular outcomes in depressed versus control pregnancies.

Outcome	Depressed (*n* = 130)	Controls (*n* = 390)	*p*-Value
Gestational hypertension	24 (18.5%)	39 (10.0%)	0.010
Preeclampsia	11 (8.5%)	19 (4.9%)	0.12
Clinically significant Holter-confirmed arrhythmia	20 (15.4%)	27 (6.9%)	0.003

**Table 3:** Crude and adjusted odds ratios for cardiovascular outcomes associated with antenatal depression.

Outcome	Crude OR	Crude 95% CI	Adjusted OR	Adjusted 95% CI	*p*-Value
Gestational hypertension	2.04	1.17–3.54	1.85	1.12–3.05	0.016
Preeclampsia	1.80	0.84–3.90	1.63	0.78–3.40	0.19
Clinically significant Holter-confirmed arrhythmia	2.44	1.32–4.53	2.05	1.18–3.57	0.011

**Table 4:** Cardiovascular outcomes stratified by depression severity.

Outcome	Mild (*n* = 44)	Moderate (*n* = 62)	Severe (*n* = 24)	*p* for Trend
Gestational hypertension	6 (13.6%)	12 (19.4%)	6 (25.0%)	0.238
Preeclampsia	2 (4.5%)	6 (9.7%)	3 (12.5%)	0.230
Holter-confirmed arrhythmia	4 (9.1%)	10 (16.1%)	6 (25.0%)	0.081

## Data Availability

The original contributions presented in this study are included in the article/supplementary material. Further inquiries can be directed to the corresponding authors.

## Supplementary Materials

The following supporting information can be downloaded at: https://www.mdpi.com/article/10.3390/jcm15113995/s1, Supplementary Table S1. Holter-confirmed arrhythmia subtypes by exposure group.

## Abbreviations

AF	Atrial fibrillation
ANS	Autonomic Nervous System
aOR	Adjusted Odds Ratio
BMI	Body Mass Index
CI	Confidence Interval
CRP	C-reactive protein
DSM-5	Diagnostic and Statistical Manual of Mental Disorders, Fifth Edition
ECG	Electrocardiography
EPDS	Edinburgh Postnatal Depression Scale
GDPR	General Data Protection Regulation
HAM-D	Hamilton Depression Rating Scale
HF	High-frequency power
HPA	Hypothalamic–Pituitary–Adrenal
HRV	Heart Rate Variability
ICD	International Classification of Diseases
IL-6	Interleukin-6
LF/HF	Low-frequency/high-frequency ratio
OR	Odds Ratio
PlGF	Placental Growth Factor
PPHN	Persistent Pulmonary Hypertension of the Newborn
PVC	Premature ventricular complex
QTc	Corrected QT interval
SCID-5	Structured Clinical Interview for DSM-5 Disorders
sEng	Soluble Endoglin
sFlt-1	Soluble fms-like tyrosine kinase-1
SNRI	Serotonin-Norepinephrine Reuptake Inhibitor
SSRI	Selective Serotonin Reuptake Inhibitor
STROBE	Strengthening the Reporting of Observational Studies in Epidemiology
SVT	Supraventricular tachycardia
TNF-alpha	Tumor necrosis factor-alpha
TTS	Takotsubo syndrome
