# Supplementary material for: Depression as a Cardiovascular Risk Marker in Pregnancy: Hypertensive and Arrhythmic Maternal Outcomes in a Retrospective Matched Cohort
Source: J Clin Med. 2026 May 22;15(11):3995. doi: 10.3390/jcm15113995 (PMC13257694; doi:10.3390/jcm15113995)
Supplement: Supplementary file 1 [file jcm-15-03995-s001.zip › jcm-4310541-supplementary.pdf]

Supplementary Table S1. Holter-confirmed arrhythmia subtypes by exposure group.

Arrhythmia Subtype	Depressed Pregnancies (n = 20)	Controls (n = 27)	Operational Definition
Supraventricular tachycardia	5	6	Holter-confirmed paroxysmal SVT or sustained supraventricular run judged clinically significant
High-burden or multifocal PVCs	3	4	>500 PVCs/24 h, >1% total beats, or multifocal PVCs
Atrial fibrillation/flutter	10	13	Any Holter-confirmed AF or atrial flutter episode
Non-sustained ventricular tachycardia	1	2	Three or more consecutive ventricular beats lasting <30 s
Clinically relevant bradyarrhythmia	1	2	Symptomatic or clinically managed bradyarrhythmia documented on Holter

The table was added to satisfy arrhythmia subtype transparency. Subtype counts were kept descriptive because individual rhythm categories were too sparse for reliable subtype-specific regression. The composite model therefore reflects clinically significant Holter-confirmed rhythm morbidity as a group, and the result should not be generalized to atrial fibrillation, supraventricular tachycardia, ectopy, ventricular tachycardia, or bradyarrhythmia separately. The number of patients who underwent Holter monitoring in each exposure group is reported separately above because the denominator for arrhythmia detection differs from the full matched cohort when monitoring is clinically triggered.
